# T-cell epitopes predicted from the Nucleocapsid protein of Sin Nombre virus restricted to 30 HLA alleles common to the North American population

**DOI:** 10.6026/97320630013094

**Published:** 2017-03-31

**Authors:** Sathish Sankar, Mageshbabu Ramamurthy, Balaji Nandagopal, Gopalan Sridharan

**Affiliations:** 1 Sri Sakthi Amma Institute of Biomedical Research, Sri Narayani Hospital and Research Centre, Sripuram, Vellore 632 055, Tamil Nadu,India;

**Keywords:** T cell epitopes, Hantaviruses, Sin Nombre;, nucleocapsid, MHC

## Abstract

Hantavirus cardiopulmonary syndrome in North America is caused by Sin Nombre virus (SNV) and poses a public health problem. We identified T-cell epitopes restricted to HLA alleles commonly seen in the N. American population. Nucleocapsid (N) protein is 428 aminoacid in length and binds to RNA and functions also as a key molecule between virus and host cell processes. The predicted epitopes from N protein that bind to class I MHC were analyzed for human proteasomes cleavage, TAP efficiency, immunogenicity and antigenicity. We identified 8 epitopes through MHC binding prediction, proteasomal cleavage prediction and TAP efficiency. Epitope VMGVIGFSF had highest Vaxijen score and the epitope, TNRAYFITR had highest immunogenicity score. Epitope AAVSALETK and TIACGLFPA had 100% homology to many HCPS causing viruses. Our study focused on T-cell epitope prediction specific to restricted HLA haplotypes of racial groups in North America for the potential vaccine development. Among the candidate epitopes, FLAARCPFL was conserved in SNV, which is suitable for vaccine specific to the virus genotype. Peptide-based vaccines can be designed to include multiple determinants from several hantavirus genotypes, or multiple epitopes from the same genotype. Thereby, immune response will focus solely on relevant epitopes, avoiding non-protective responses or immune evasion. The other advantages include absence of infectious material unlike in live or attenuated vaccines. There is no risk of reversion or formation of adverse reassortants leading to virulence and no risk of genetic integration or recombination forming a rationale for vaccine design including for distinct geographical regions.

## Background

Sin Nombre virus (SNV) belongs to Hantavirus genus (Family Bunyaviridae). Goldsmith et al. [1] documented the virus morphology using electron microscopy and immunoelectron microscopy. It is the causative agent of hantavirus cardiopulmonary syndrome (HCPS) in humans transmitted by its rodent reservoir, North American deer mouse (Peromyscus maniculatus). Chizhikov et al. [2] reported the complete genetic characterization of SNV and the exact 5'- and 3'- terminal sequences of the three genomic segments. Remote sensing and geographic information system maps of SNV infections in deer mouse populations has been documented by Boone et al. [3]. A relationship between host density and infection dynamics was studied [4]. Terajima and Ennis [5] reported the quantitative measurement of viral RNA in human samples. They indicated that antibody-bound viruses and unbound viruses were measurable by quantitative RT-PCR. SNV persists to be the predominant hantavirus causing HCPS in the United States [6] and Canada [7]. As of January 2016, 659 HCPS cases have been reported with the case fatality rate of 36% in USA (http://www.cdc.gov/hantavirus/surveillance/annualcases. html).

Ye et al. [8] reported the presence of high titers of neutralizing antibodies months after recovery. Nucleocapsid (N) protein coded by S segment of the virus genome has been used for diagnosis due to its antigenic properties [9]. The amino and carboxy termini of the N protein are inferred to form trimers in the protein generation [10]. Diagnoses by PCR testing for specific and pan-hantaviruses have been reported. There is no specific antiviral treatment option available but only supportive therapy and blood oxygenation. Minimizing or eliminating contact with rodents to help prevent exposure to the virus could prevent this condition. Safronetz et al. [11] and Brocato et al. [12] have successfully used animal models to establish persistent infection in which it may be possible to test antiviral agents and vaccines. Vaccines against SNV are still under development for use to avoid outbreaks [13].

The other fatal infection caused by hantaviruses is hemmorhagic fever with renal syndrome (HFRS). This is seen predominantly in Asian countries. Increased vascular permeability and leakage in the kidneys and the lungs are responsible for characteristic difference in the respective disease caused by the different genotypes. A prophylactic T cell epitope based vaccine could induce CTL immunity, which will protect against viral disease like in the case of Dengue virus [14].

As in the case of vaccine preventable viral diseases, preexisting T and B cell immunity could avert disease. Good CD4 T cell priming by peptide vaccination could improve antibody response also during natural infection that could occur in the immunized individuals, "primed by vaccines, boosted by natural infection" is a good vaccine strategy [15]. Infection could occur in vaccinated individuals, but no disease is seen, in the case of killed poliovirus vaccine, even gut infection by poliovirus is prevented [16].

The increased understanding of antigen recognition at molecular level has resulted in the development of rationally designed peptide vaccines. In the present study, we used immunoinformatics strategies for designing vaccine candidate Tcell epitopes. These peptide’s epitopes are important towards development of T-cell epitope-based vaccines that could bind to specific Class I MHC and thereby stimulate T-cell immune responses.

We aimed to identify candidate T-cell epitopes of SNV that are restricted to HLA alleles common to North American population where this virus is widespread. The epitopes that bind to Class I MHC that is also cleaved at the flanking regions by human proteasomes and transporter associated with antigen processing (TAP) efficiency was also analyzed.

## Methodology

### Retrieval of nucleotide sequences

All available complete S segment amino acid (aa) sequences (n=11) of strains of Sin Nombre virus that causes Hantavitus Cardiopulmonary Syndrome were retrieved from GenBank database [17] as of October 2016. A consensus aa sequence was identified using CLC sequence Viewer 7 program (https://www.qiagenbioinformatics.com/). The program identifies the consensus sequence based on most frequent residues found at each position in the sequence alignment. The consensus sequence was used for further analysis to identify Tcell epitopes.

### Selection of MHC alleles

We selected the top 30 human Class I MHC alleles reported for Whites, Blacks, Hispanics and Asian or Pacific Islander population groups of the North American population [18]. The selected alleles were based on the percentage chance of haplotype expressed in an individual identified from HLA matchmaker program available at http://www.epitopes.net/.

### Prediction of epitopes from the N protein of Sin Nombre virus with affinity to Class I MHC molecules

Using the identified consensus aa sequences as the input, T-cell epitopes that bind to MHC Class I were predicted using NetMHCpan 3.1 online server. This program predicts binding of peptides to any MHC molecule of known sequence using artificial neural networks (ANNs) [19]. The epitopes of 9-mer and 10-mer lengths were derived. The program also had a wide choice of alleles to choose and select as a query. HLA alleles that occur most commonly in the North American population were selected for epitope identification. The default threshold for strong binding and weak binding in terms of % rank, 0.5 and 2 respectively was used in our study as in previous reports on other analytical approaches. Strong binders alone were selected and used for further analysis.

### Prediction of proteasomal cleavage

This was predicted using MAPPP (MHC-I Antigenic Peptide Processing Prediction) program [20]. The program generates a probability for the cleavage of each possible peptide from a protein by the proteasome in the cell and the probability is based on a statistic-empirical method. The algorithms in the program were earlier implemented in FRAGPREDICT. Minimum possibility for cleavage after a single residue and for cleavage of a fragment was set to default value of 0.5.

### Prediction of TAP efficiency

To predict the candidate epitope(s) based on the processing of the peptide(s) in vivo, the transporter of antigenic peptides (TAP) proteins' transport efficiency was tested using TAPPred server program [21]. The prediction approach used in this study was cascade Support Vector Machines (SVM), a prediction that is based on the sequence and features of amino acids and their properties.

### Prediction of antigenicity/immunogenicity

The identified epitope(s) were used to predict whole protein antigenicity (protective antigen) using Vaxijen 2.0 server program with a threshold limit of 0.5 [22]. The threshold values of the highest accuracy of more than 0.5 were considered probable antigens and were selected for further analysis. In addition, class I immunogenicity analysis was carried out in an online server tool available at http://tools.iedb.org/immunogenicity/. This tool uses amino acid properties as well as their position within the peptide to predict the immunogenicity of a peptide MHC (pMHC) complex.

## Results

A consensus sequence of Sin Nombre virus of length 428 aa was identified from all available complete S segment sequence coding for nucleocapsid protein (N protein). A total of 120 HLA types were selected for their preponderance in the North American racial groups. Analysis for epitopes restricted to specified class I MHC resulted in 478 possible epitopes [HLA-A* (n=171), HLA-B* (n=146) and HLA-C* (n=161)]. The results are presented in Table 1.

Common HLA alleles were found in the four groups of North American population and many common T-cell epitopes were identified from different HLA alleles due to promiscuous presentation of the same T-cell epitope via two or more HLA class I molecules. Therefore, a non-redundant 63 HLA alleles [HLA-A* (n=21), HLA-B* (n=25) and HLA-C* (n=17)] was generated and epitope dataset (n=85) were identified restricted to these alleles. Among the top 30 alleles in North American population, alleles A*02:01, B*44:03, C*03:04, C*04:01, C*06:02, C*07:02 were present in all four population groups.

TAPpred analysis was carried out using full-length consensus amino acid sequence of Sin Nombre nucleocapsid coding protein. The analysis resulted in 420 possible epitopes with varying affinities classified as high (n=164), intermediate (186) and low or detectable (n=70). A total of 47 epitopes were identified both by NetMHCpan 3.0 and TAPpred programs. These epitopes were analyzed for proteasome cleavage analysis.

Further screening based on proteasome cleavage resulted in 8 epitopes with scores ranging from 0.5009 to 1 (Table 2). Among them, six have been identified as probable antigen by Vaxijen program and were further analyzed for immogenicity. Epitopes VMGVIGFSF had highest Vaxijen score of 1.8515 followed by AAVSALETK (1.5281), FLAARCPFL (1.2043), QSRRAAVSA (0.8992), TNRAYFITR (0.6425), and TIACGLFPA (0.597). Among the epitopes, TNRAYFITR had highest immunogenicity score (0.29777) followed by VMGVIGFSF (0.21618), QSRRAAVSA (0.08199) and TIACGLFPA (0.07333).

Epitope VMGVIGFSF was predicted to be restricted to bind in the binding groove of 5 HLA types viz. A*23:01, A*24:02, A*32:01, B*15:01 and B*15:03. These types are spread in one or other of four population groups. Protein BLAST analysis of the epitope resulted in 100% homology to Puumala virus that causes milder Nephropathia epidemica, and Khabarovsk virus that causes HFRS suitable for multigenotype vaccine.

Epitope AAVSALETK is restricted only to A*11:01. These alleles are widespread in Whites, Hispanics and Pacific Islander population groups. BLAST analysis of this epitope resulted in 100% homology to many HCPS causing viruses: Convict Creek Canal virus, Bayou virus, New York virus, Montano virus, Jabora virus, and El Moro Canyon virus in addition to Sin Nombre virus.

Epitope FLAARCPFL was predicted to bind to 9 different HLA alleles A*02:01, A*02:03, A*02:06, A*02:07, B*08:01, C*01:02, C*04:01, C*17:01, and C*18:01. Of these, A*02:01 and C*04:01 was present in all four population groups. Other 7 alleles are present in one or other population groups. BLAST analysis of this epitope resulted in 100% homology to Sin Nombre virus.

Epitope QSRRAAVSA was restricted to HLA A*30:01 that is present only in top 30 alleles of black population groups. Epitope TNRAYFITR was predicted to bind to HLA A*31:01 and A*33:03. These alleles are among the top 30 alleles of Blacks, Hispanics and Pacific Islander population groups. BLAST analysis of this epitope resulted in 100% homology to Tula virus, Bayou virus, LANV-2, Montano virus, El Moro Canyon virus, Convict Creek Canal virus in addition to Sin Nombre virus which all cause HCPS.

Epitope TIACGLFPA was restricted to HLA A*02:01, A*02:03, A*02:06, and A*68:02 spread in the four population groups. BLAST analysis of this epitope resulted in 100% homology to Andes virus, Convict Creek Canal virus, LANV-2, Araucaria virus, Choclo virus, New York virus, RIOMV-4, and Juquitiba virus which all cause HCPS.

## Discussion

Sin Nombre virus is an important etiological agent of HCPS mainly in North America [23]. Sporadic HCPS cases occur largely in rural areas where forests, fields, and farms form suitable habitat for the rodent reservoir host (the deer mouse) present throughout many parts of USA and Canada. Other viruses that potentially cause HCPS (with evidence of disease association in humans) are the New York virus, the Black Creek Canal virus, Andes virus, Laguna Negra virus (LANV-2), Rio Mamore virus (RIOMV-4), El Moro Canyon virus, Araucaria, Choclo, Araraquara, Jubiquito, Jabora, Maripa, Tunari. Among these New York virus and Black Creek Canal virus are reported to be prevalent in northeastern and southeastern USA respectively [24].

Schountz et al. [25] demonstrated the SNV dissemination in infected mice and the timeline of virus infection with antibody demonstration. The study did not look at cellular immune response. Amman et al. [26] have documented the epizootic nature of SNV. Evidence of Sin Nombre virus infection is established in several parts of USA [27], but appropriate control and prevention policies are still inadequate. A suitable vaccine may be one important tool in the control of infection. Hence, we identified candidate T-cell epitopes that are restricted to HLA alleles commonly seen in American population. The predicted epitopes that bind to class I MHC was also analyzed for human proteasomes cleavage, TAP efficiency, immunogenicity and antigenicity. This approach would facilitate geographic regionspecific pathogen directed vaccine.

HLA haplotypes of host are crucial determinants of both B and T cell specific immune response. Hooper et al. [13] have successfully shown testing of a SNV full-length M gene-based DNA vaccine in rabbits. The immunized animals showed high titers of neutralizing antibodies. CD4+ T cells recognize viral T Cell antigenic epitopes when they are located in the groove of Class I MHC molecules on non-professional antigen presenting cells (APC). Almost any infected cell, e.g. tissue fibroblasts including professional APC like macrophages and dendritic cells can present the antigenic epitopes. This is vital for an afferent T cell response which gives rise to Cytotoxic T cells (CTL) and memory T cells. B cells recognize viral epitopes presented on the professional APCs when the TAP molecules in the grooves of MHC Class II antigens locate these. CD4 helper T cell function is simultaneously involved in generating antibody producing plasma cells and memory B cells. The intensity of immune response with good immunological memory will be achieved when the epitopes have high affinity to the host MHC molecules on APC [28].

Schountz et al. [29] examined CD4+ T cell responses in mice infected with SNV. Lymphocyte proliferation responses to the N protein were weak in experimental infection. Cytokines, including IFN-γ, IL-4, IL-5, and TGF-β1, but not TNF, lymphotoxin, or IL-17 were produced in the mice. The authors conclude that TGF-β1-expression results in an inhibitory effect through regulatory T cells on host disease and viral clearance.

HLA allele sequence are very diverse belonging to six different classes (A, B, C, E, F, G), a total of 11406 alleles have been identified as of October 2016 (IPD-IMGT/HLA database release 3.26.0) [30]. Human class I MHC molecules (HLA-A, HLA-B, HLA-C) are highly polymorphic. They present antigenic peptides to the TCR expressed by CTLs. HLA polymorphism is the outcome of natural selection for achieving pathogen specific immunity [31]. The highly diverse HLA, in the human genome play an important role in host-pathogen interaction by mediating innate and adaptive cellular immune responses. HLA alleles have been associated with severity, varied disease outcome, persistence, emergence and transmission for several infectious diseases [32].

HLA molecules significantly overlap in peptide binding specificity. Class I HLA peptide binding shows a high degree (>60%) of promiscuity [33]. HLA allelic variation occurs in different ethnicities [34] and therefore must be an important consideration while designing and developing T-cell epitopebased diagnostics or vaccines, where multiple epitopes with different HLA binding specificities are screened.

HLA allele frequencies exhibit ethnic variation, with some alleles found widely distributed among populations and others almost exclusively within a particular ethnic group. The Class I and II loci reside on a relatively small region of human chromosome 6 and specific haplotypes. Apparently, they are present at high frequencies in founding populations or were selected for generating immune response to the infectious organisms. In this setting, linkage disequilibrium results in a significant over representation of certain haplotypes [35]. An ethnic and geographical difference in HLA has been shown to be associated with disease outcome, such as viral persistence or viral clearance [36]. Therefore, HLA diversity data has become increasingly important in the design of population-specific T-cellbased vaccines [37]. HLA diversity data was thus utilized suitably in our study to predict T-cell epitopes specific to the population where the infection is widespread.

Hantavirus specific CD8+ and CD4+ CTL are thought to contribute to the immunopathology and capillary leak syndrome observed in the HCPS [38]. Kilpatrick et al. [39] identified three CD8+ T cell epitopes in SNV presented by HLA-B*35:01 and quantitated circulating SNV-specific CD8+ T cells in 11 acute HPS patients using HLA/peptide tetramers. Individuals with HLAB* 35:01 had an increased risk of developing severe HCPS, suggesting that CD8+ T cell responses to SNV contribute to pathogenesis.

The present approach is to use peptide sequence data for experimental determination of affinity. Such findings have been used in the construction of many T-cell epitope prediction algorithms and the outcome of such analysis is robust [40]. However, previously, HLA diversity for a given population was not considered while developing vaccines.

Conventional experimental HLA typing using next generation sequencing tool and mapping an optimal CD8 T-cell epitope is laborious and expensive. Now, bioinformatic tools have been developed that predict peptides that bind to a specific MHC molecule. Though the experimental fine mapping of epitopes are unmatched in their efficacy [41]. Prediction methods also are equally indispensable to experimental validation methods for better vaccine development [42].

The application of information from the fields of pharmacogenomics, pharmacogenetics and bioinformatics to vaccine design termed ‘vaccinomics’ has potential advantages. The conventional experimental approaches are seen as a bottleneck toward developing new vaccines simply because of the possibility of potential candidate epitopes being left unnoticed. Availability of pathogen genomes is now the key wealth of information and the computer programs developed with extremely powerful algorithms can handle even a huge dataset for informatics-based approach towards vaccine design. Moreover, possibility of T-cell epitope prediction that bind to specific HLA-class/allele, transporter of antigen processing (TAP) affinity prediction and proteasomal cleavage prediction are highly beneficial. Screening peptide-based vaccines using in silico bioinformatic approach has been shown to be particularly useful when hyper variable viruses like HIV and HCV are examined [43]. We also believe that this applies to hantaviruses as well simply because they are very diverse and causing different clinical syndromes in different areas and each transmitted by different rodent hosts. Ample choices of T-cell epitopes identified through these bioinformatic approaches can be developed into a synthetic polyvalent peptide vaccines suitable for diverse HLA types in each population.

In the course of Class I MHC presentation, antigens that are synthesized in the cytosol undergo proteasomal degradation and Transporter associated with Antigen Presentation (TAP) molecules [44] transports the generated peptides into the endoplasmic reticulum (ER). Inside the ER, the peptides bind to Class I MHC molecules, and carried to the cell surface. The MHCI and peptide complex are then recognized by CTLs. Cytotoxic T cells encounter smaller peptides (eight to ten amino acids) in length. Peters et al. [45] reported that combining in silico predictions of MHC-I binding affinities along with predictions of TAP transport efficiency lead to an improved identification of epitopes, compared to predictions of MHC-I binding affinities combined with predictions of C-terminal cleavages made by the proteasome. Nevertheless, the proteasome system plays an important role in MHC Class I antigen processing and presentation [46] and as a result activation of CD8+ T cells, as well as activation of the NF-κB pathway [47] for mounting immune response. Ip et al. [48] reported that the prediction of MHC class I epitopes for HCV and proteasomal cleavage sites prediction at the flanking regions of epitopes enhances the precision of identification of functional HCV-specific CTL epitopes. In our study, we screened for T-cell epitopes for potential vaccine candidate using bioinformatic approaches integrating both proteasome cleavage prediction and TAP affinity prediction along with antigenic and immunogenic abilities. This significantly improves the strength of prediction ability for further evaluation in animal models and finally in human population.

**Figure 2:**
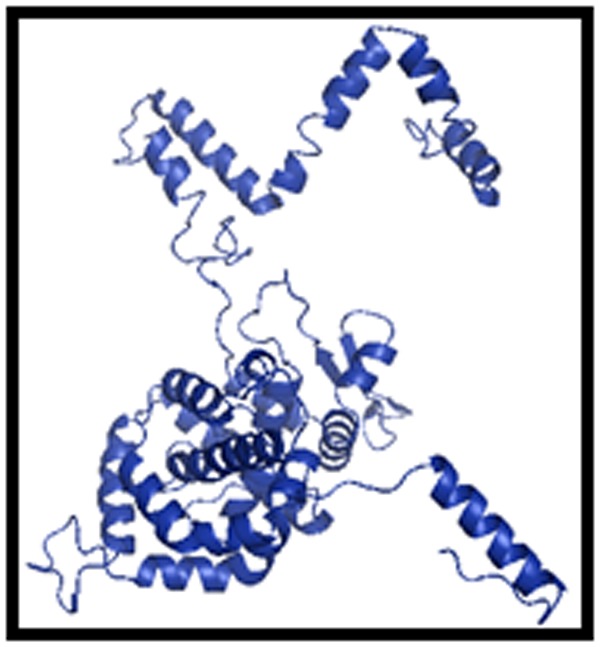
3D structure of SNV N protein generated by I-TASSER program

Previously, we had demonstrated immunodominant B-cell epitope of SNV in the N protein [49]. The 3D structure generated using I-TASSER program is shown in Figure 2. In our study, the generated candidate T-cell epitopes (9-mer and 10-mer) ranged from three to thirteen specific to each allele. No epitope was identified for HLA-A*29:02 by the program. The NetMHCpan 3.0 program used in our study is based on neural network-based machine-learning algorithm. This allows insertions and deletions in a pan-specific MHC-I binding machine-learning model and also enables combining information across both multiple MHC molecules and peptide lengths. The above pan-allele/pan-length algorithm is a state-of-the-art method with increased accuracy for ligand identification [50].

MAPPP, which stands for MHC-I Antigenic Peptide Processing Prediction, predicts proteasomal cleavage with peptide anchoring to MHC I molecules. This program accepts length of fragments between 9 and 11. Though a TAP transporter can translocate peptides of 8-40 amino acids, with preference for peptides of length 8 to 11 amino acids, many programs including TAPpred used in our study predicts nonamers (9-mer) only. Therefore, 10- mer epitopes predicted in MHC binding program and MAPPP program, were eliminated in the TAP efficiency analysis. Due to this reason, the finalized epitopes were all nonamers.

The steps of MHC class I antigen presentation pathway are evaluated by three scoring systems. 1. proteasomal score which reflects the efficiency of antigen-processing examining cleavage site usage releasing the peptide C-terminus. 2. TAP score predicts transporter molecule associated with the epitope transport. This is achieved by estimation of the binding of a given peptide to TAP. The highest affinity score for a peptide indicates the highest transport rates and affinity for the MHC molecule. The scores are expressed logarithmically; higher values indicate higher predicted efficiency.

Following this, the identification of variables that influence immunogenicity has also been identified as an important step in the investigation of T-cell epitopes and understanding of cellular immune responses [51]. In the immunogenicity analysis program we used, positions P4-6 of a presented peptide and amino acids with large and aromatic side chains, which are associated with immunogenicity are taken into consideration. Also, in this program, T-cells are equipped to better recognize viral than human (self) peptides. Similarly, Vaxijen model for prediction of protective viral antigens was used. The model was reported to have prediction accuracy up to 89% [52].

Highlights of our study include T-cell epitope prediction specific to geographically restricted HLAs for the potential vaccine development for hantavirus infection. Among the candidate epitopes identified in our study, FLAARCPFL was conserved in Sin Nombre virus, which is suitable for a vaccine specific to this virus genotype. Other epitopes were conserved across the HCPS causing hantaviruses suitable for pan-hantavirus vaccine. The data generated in this study has an intriguing potential for more rational approaches for vaccine design. SNV continues to be a significant cause of morbidity and mortality in N. America and its control is not possible because of several epidemiological features and lack of specific therapy. Development and application of an effective vaccine may be one important approach to be explored for the control of SNV infection.

## Conflict of Interest

None

## Figures and Tables

**Table 1:** List of T-cell epitopes with strong binding affinity to MHC Class I alleles (SB: strong binders)

HLA types	Epitopes (SB)									
HLA-A										
A*01:01	HLKEKSSLRY	TADWKSIGLY	QLDQKIIILY							
A*02:01	YILSFALPII	MGVIGFSFFV	ALYVAGMPEL	GLYILSFAL	YILSFALPI	ILSFALPII	TIACGLFPA	GVIGFSFFV	FLAARCPFL	
A*02:03	ALYVAGMPEL	YMSHWGREAV	ILSFALPII	HLYVSMPTA	TIACGLFPA	NIISPVMGV	FLAARCPFL	YMSHWGREA		
A*02:06	YILSFALPII	RTIACGLFPA	MGVIGFSFFV	YILSFALPI	IILKALYML	IACGLFPAQ	NIISPVMGV	GVIGFSFFV	FLAARCPFL	
A*02:07	IGLYILSFAL	YILSFALPII	ILSFALPIIL	MGVIGFSFFV	DFLAARCPFL	ALYVAGMPEL	GLYILSFAL	YILSFALPI	ILSFALPII	IILKALYML
	GVIGFSFFV	FLAARCPFL								
A*03:01	LIAAQKLASK	LSFALPIILK	SMPTAQSTMK	GVIGFSFFVK	VIGFSFFVK	KLKKKSAFY				
A*11:01	LSFALPIILK	GVIGFSFFVK	ATNRAYFITR	KSAFYQSYLR	QSMGIQLDQK	AAVSALETK	VIGFSFFVK	SAFYQSYLR		
A*23:01	LYILSFALPI	RFRTIACGLF	VMGVIGFSF	LYVAGMPEL	SYLRRTQSM					
A*24:02	LYILSFALPI	RFRTIACGLF	KDWMERIDDF	VMGVIGFSF	ATPHSVWVF	LYVAGMPEL	SYLRRTQSM	DAALATNRAY		
A*25:01	DAALATNRAY	NIISPVMGV	FVKDWMERI	ESATIFADI	DIATPHSVW	NTIMASKSV				
A*26:01	EVQDNITLH	FVKDWMERI								
A*29:02	-	-	-	-	-	-	-	-	-	-
A*30:01	GIRKPRHLYV	RTIACGLFPA	VKARNIISPV	KARNIISPVM	QSRRAAVSA	KSSLRYGNV	STRGRQTIK	RFRTIACGL	KARNIISPV	
A*30:02	TADWKSIGLY	RIRFKDDSSY	GIRKPRHLY	AALATNRAY	KLKKKSAFY					
A*31:01	SFFVKDWMER	ATNRAYFITR	AFFAILQDMR	KSAFYQSYLR	SAFYQSYLRR	IILYMSHWGR	HLKEKSSLR	KALYMLSTR	LYMLSTRGR	RIDDFLAAR
	TNRAYFITR	SAFYQSYLR	AFYQSYLRR	ILYMSHWGR						
A*32:01	KSIGLYILSF	GLYILSFAL	YILSFALPI	IILKALYML	VMGVIGFSF	RAYFITRQL	KSAFYQSYL	RTQSMGIQL		
A*33:03	SFFVKDWMER	KSAFYQSYLR	SAFYQSYLRR	IILYMSHWGR	HLKEKSSLR	LYMLSTRGR	EVNGIRKPR	FFVKDWMER	TNRAYFITR	FFAILQDMR
	SAFYQSYLR	ILYMSHWGR								
A*34:02	LIAAQKLASK	LSFALPIILK	GVIGFSFFVK	DMRNTIMASK	SAFYQSYLRR	QSMGIQLDQK	IAAQKLASK	SAFYQSYLR		
A*68:01	ELADLIAAQK	GVIGFSFFVK	KSAFYQSYLR	SAFYQSYLRR	EVNGIRKPR	WVFACAPDR	SAFYQSYLR			
A*68:02	QTADWKSIGL	MGVIGFSFFV	ESATIFADIA	TIACGLFPA	NIISPVMGV	GVIGFSFFV	ESATIFADI	HSVWVFACA	NTIMASKSV	MSHWGREAV
A*74:01	ALYMLSTRGR	GLFPAQVKAR	GVIGFSFFVK	ATNRAYFITR	KSAFYQSYLR	SAFYQSYLRR	IILYMSHWGR	KALYMLSTR	VIGFSFFVK	RIDDFLAAR
	SAFYQSYLR	ILYMSHWGR								
HLA-B										
B*07:02	RKPRHLYVSM	KPRHLYVSMP	TPGRFRTIAC	KARNIISPVM	KDPRDAALAT	APDRCPPTAL	MPELGAFFAI	KPRHLYVSM	TPGRFRTIA	KARNIISPV
B*08:01	MLSTRGRQTI	ILQDMRNTIM	TLQSRRAAV	KPRHLYVSM	FLAARCPFL	SYLRRTQSM				
B*13:01	AESATIFADI	MPELGAFFAI	RELAQTLVDI	KEVQDNITL	YILSFALPI	PELGAFFAI	REAVNHFHL	REISNQEPL		
B*14:02	NRAYFITRQL	SRRAAVSAL	DHLKEKSSL	SYLRRTQSM						
B*15:01	KSIGLYILSF	VMGVIGFSFF	ALATNRAYF							
B*15:02	AALATNRAY									
B*15:03	IRFKDDSSY	VMGVIGFSF	AALATNRAY	ARAESATIF	LQDMRNTIM	KKSAFYQSY				
B*18:01	DDFLAARCPF	MPELGAFFAI	HEQQLVTAR							
B*35:01	DAALATNRAY	MPELGAFFAI	LPIILKALY	SPVMGVIGF	AALATNRAY	MPELGAFFA				
B*38:02	MPELGAFFAI	WKSIGLYIL	NHFHLGDDM							
B*40:01	LKEVQDNITL	AESATIFADI	MPELGAFFAI	GREAVNHFHL	RELAQTLVDI	VREISNQEPL	KEVQDNITL	PELGAFFAI	REAVNHFHL	REISNQEPL
B*40:02	AESATIFADI	MPELGAFFAI	RELAQTLVDI	KEVQDNITL	RELADLIAA	PELGAFFAI	REAVNHFHL	REISNQEPL		
B*42:01	LPIILKALYM	RKPRHLYVSM	TPGRFRTIAC	FPAQVKARNI	APDRCPPTAL	MPELGAFFAI	KPRHLYVSM	TPGRFRTIA		
B*44:02	EEPSGQTADW	KENKGTRIRF	AESATIFADI	ADIATPHSVW	MPELGAFFAI					
B*44:03	EEPSGQTADW	AESATIFADI	ADIATPHSVW	MPELGAFFAI						
B*45:01	KRELADLIAA	MERIDDFLAA	AESATIFADI	MPELGAFFAI	RELADLIAA	MERIDDFLA	AESATIFAD			
B*46:01	FALPIILKAL	VSMPTAQSTM	YILSFALPI	MGVIGFSFF	FSFFVKDWM					
B*49:01	AESATIFADI	MPELGAFFAI	RELAQTLVDI	REAVNHFHL						
B*51:01	FPAQVKARNI	MPELGAFFAI	YILSFALPI	LATNRAYFI	IATPHSVWV					
B*52:01	MPELGAFFAI	RELAQTLVDI	YILSFALPI	LSFALPIIL	RAYFITRQL	IQLDQKIII				
B*53:01	LPIILKALYM	FPAQVKARNI	MPELGAFFAI	EPSGQTADW	LPIILKALY	SPVMGVIGF				
B*54:01	MPTAQSTMKA	SPVMGVIGFS	ATPHSVWVFA	TPHSVWVFAC	MPELGAFFAI	FALPIILKA	TPHSVWVFA	CPPTALYVA	MPELGAFFA	
B*55:02	MPTAQSTMKA	ATPHSVWVFA	TPHSVWVFAC	MPELGAFFAI	TPGRFRTIA	TPHSVWVFA	CPPTALYVA	MPELGAFFA		
B*57:01	KSIGLYILSF	IGFSFFVKDW	IATPHSVWVF	KIIILYMSHW	KSAFYQSYL	IIILYMSHW				
B*58:01	KSIGLYILSF	IATPHSVWVF	KIIILYMSHW	LSFALPIIL	KSAFYQSYL					
HLA-C										
C*01:02	SSLRYGNVL	VLDVNSIDL	TADWKSIGL	YILSFALPI	SMPTAQSTM	ITPGRFRTI	FLAARCPFL	RAYFITRQL	KSAFYQSYL	RTQSMGIQL
C*02:02	FADIATPHSV	LSFALPIIL	IISPVMGVI	FSFFVKDWM	FVKDWMERI	RAYFITRQL	IATPHSVWV	KSAFYQSYL	MSHWGREAV	
C*03:02	FALPIILKAL	YILSFALPI	LSFALPIIL	FSFFVKDWM	AALATNRAY	RAYFITRQL	MSHWGREAV			
C*03:03	FALPIILKAL	FADIATPHSV	SSLRYGNVL	YILSFALPI	LSFALPIIL	RAYFITRQL	IATPHSVWV	MSHWGREAV		
C*03:04	FALPIILKAL	FADIATPHSV	SSLRYGNVL	YILSFALPI	LSFALPIIL	RAYFITRQL	IATPHSVWV	MSHWGREAV		
C*04:01	VLDVNSIDL	FRTIACGLF	WMERIDDFL	FLAARCPFL	GMPELGAFF	LQDMRNTIM	RTQSMGIQL			
C*05:01	LADLIAAQKL	KADEITPGRF	FADIATPHSV	VLDVNSIDL	TADWKSIGL	WMERIDDFL	LQDMRNTIM	KSAFYQSYL	RTQSMGIQL	LGDDMDPEL
C*06:02	NRAYFITRQL	YLRRTQSMGI	SRRAAVSAL	LRYGNVLDV	IRKPRHLYV	FRTIACGLF	ARNIISPVM	FVKDWMERI	RAYFITRQL	YFITRQLQV
	ARAESATIF	SYLRRTQSM	LRRTQSMGI							
C*07:01	NRAYFITRQL	YLRRTQSMGI	SRRAAVSAL	LRYGNVLDV	IRKPRHLYV	FRTIACGLF	ARNIISPVM	RAYFITRQL	YFITRQLQV	ARAESATIF
	SYLRRTQSM	LRRTQSMGI								
C*07:02	SRRAAVSAL	LRYGNVLDV	IRKPRHLYV	FRTIACGLF	ARNIISPVM	RAYFITRQL	YFITRQLQV	ARAESATIF	LYVAGMPEL	SYLRRTQSM
C*08:01	FALPIILKAL	FADIATPHSV	TADWKSIGL	YILSFALPI	LSFALPIIL	FALPIILKA	FSFFVKDWM	WMERIDDFL	RAYFITRQL	IATPHSVWV
	MSHWGREAV									
C*08:02	LADLIAAQKL	FADIATPHSV	VLDVNSIDL	TADWKSIGL	WMERIDDFL	LQDMRNTIM	MSHWGREAV	LGDDMDPEL		
C*12:03	FADIATPHSV	YILSFALPI	LSFALPIIL	FALPIILKA	SSYEEVNGI	KARNIISPV	FSFFVKDWM	FVKDWMERI	LATNRAYFI	RAYFITRQL
	IATPHSVWV	MSHWGREAV								
C*14:02	SRRAAVSAL	SMPTAQSTM	RFRTIACGL	RAYFITRQL	YFITRQLQV	LYVAGMPEL	AFFAILQDM	SYLRRTQSM		
C*16:01	YILSFALPI	LSFALPIIL	FSFFVKDWM	RAYFITRQL	KSAFYQSYL	MSHWGREAV				
C*17:01	FADIATPHSV	TADWKSIGL	LSFALPIIL	IISPVMGVI	WMERIDDFL	FLAARCPFL	LATNRAYFI	RAYFITRQL	KVSDIEDLI	IATPHSVWV
	KSAFYQSYL	RTQSMGIQL	MSHWGREAV							
C*18:01	SRRAAVSAL	IRKPRHLYV	FRTIACGLF	ARNIISPVM	FLAARCPFL	YFITRQLQV	ARAESATIF	LQDMRNTIM	SYLRRTQSM	

**Table 2:** List of Class I MHC T-cell epitopes with their predicted proteasome cleavage, TAP efficiency, antigenicity and immunogenicity

List of epitopes	Peptide Rank	Start Position	Proteasome score*	TAP Score and its predicted affinity*	Vaxijen score*	Immunogenicity score*
AAVSALETK	286	49	0.5009	3.907 (Intermediate)	1.5281 (Probable antigen)	-0.01823
FLAARCPFL	218	239	0.9323	4.846 (Intermediate)	1.2043 (Probable antigen)	0.11076
KARNIISPV	291	211	0.6363	3.89 (Intermediate)	0.0035 (Probable non-antigen)	0.11907
LYVAGMPEL	236	319	1	4.602 (Intermediate)	0.3162 (Probable non-antigen)	-0.03039
QSRRAAVSA	174	45	0.5548	5.81 (Intermediate)	0.8992 (Probable antigen)	0.08199
TIACGLFPA	52	200	0.5077	8.202 (High)	0.5097 (Probable antigen)	0.07333
TNRAYFITR	299	260	0.5741	3.863 (Intermediate)	0.6425 (Probable antigen)	0.29777
VMGVIGFSF	240	219	0.5015	4.523 (Intermediate)	1.8515 (Probable antigen)	0.21618

**Figure 1:**
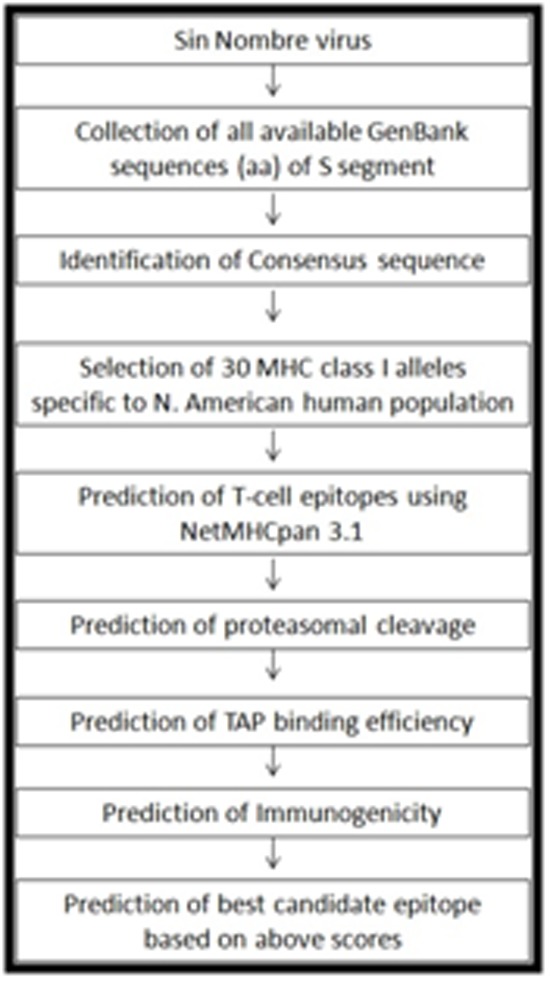
Flowchart indicating the study design

## References

[1] Goldsmith CS (1995). Arch Virol..

[2] Chizhikov VE (1995). J Virol..

[3] Boone JD (2002). Am J Trop Med Hyg..

[4] Boone JD (2000). Emerg Infect Dis..

[5] Terajima M (2003). J Virol Methods..

[6] Knust B, Rollin PE. (2013). Emerg Infect Dis..

[7] Webster D (2007). Am J Trop Med Hyg..

[8] Ye C (2004). Emerg Infect Dis..

[9] Yoshimatsu K, Arikawa (2014). J. Virus Res.

[10] Boudko SP (2007). J Mol Biol..

[11] Safronetz D (2013). J Virol..

[12] Brocato RL (2014). J Virol..

[13] Hooper JW (2013). Vaccine..

[14] Comber JD (2014). Hum Vaccin Immunother.

[15] Ciabattini A (2013). Front Immunol..

[16] http://www.globalvaccines.org.

[17] http://www.ncbi.nlm.nih.gov/nucleotide.

[18] http://www.proimmune.com/ecommerce/page.php?page=MHC_alleles.

[19] http://www.cbs.dtu.dk/services/NetMHCpan/.

[20] http://www.mpiib-berlin.mpg.de/MAPPP/cleavage.html.

[21] http://www.imtech.res.in/raghava/tappred/.

[22] http://www.ddg-pharmfac.net/vaxijen/VaxiJen/VaxiJen.html.

[23] Richardson KS (2013). Ecohealth.

[24] http://www.cdc.gov/hantavirus/hps/transmission.html.

[25] Schountz T (2012). J Virol..

[26] Amman BR (2013). J Wildl Dis..

[27] Núñez JJ (2014). Emerg Infect Dis..

[28] Li Pira G (2007). Cytometry B Clin Cytom..

[29] Schountz T (2007). Proc Natl Acad Sci U S A..

[30] http://hla.alleles.org/nomenclature/stats.html.

[31] Archbold JK (2009). J Exp Med..

[32] Harris RA (2008). Hepatology..

[33] Rao X (2011). Immunogenetics.

[34] Maiers M (2007). Hum Immunol.

[35] Sanchez-Mazas A, Meyer D.  (2014). J Immunol Res..

[36] Kawashima Y (2009). Nature..

[37] Tshabalala M (2015). J Immunol Res.

[38] Ennis FA (1997). Virology.

[39] Kilpatrick ED (2004). J Immunol.

[40] Soria-Guerra RE (2015). J Biomed Inform.

[41] Roider J (2014). Immunology.

[42] Lundegaard C (2012). Expert Rev Vaccines.

[43] Sirskyj D (2011). Immunol Cell Biol.

[44] Blum JS (2013). Annu Rev Immunol..

[45] Peters B (2003). J Immunol.

[46] Sijts EJ, Kloetzel PM. (2011). Cell Mol Life Sci.

[47] McCarthy MK, Weinberg JB. (2015). Front Microbiol.

[48] Ip PP (2015). Vaccines (Basel)..

[49] Kalaiselvan S (2017). J Cell Biochem..

[50] Nielsen M, Andreatta M (2016). Genome Med.

[51] Calis JJ (2013). PLoS Comput Biol.

[52] Doytchinova IA, Flower DR. (2007). BMC Bioinformatics.

